# Substrate Interactions and Free-Swimming Dynamics in the Crayfish Escape Response

**DOI:** 10.1093/iob/obae027

**Published:** 2024-07-12

**Authors:** L X de Pablo, A Carleton, Y Modarres-Sadeghi, E D Clotfelter

**Affiliations:** Department of Biology, Amherst College, Amherst MA 01002, USA; Present Affiliation: Department of Ecology and Evolutionary Biology, University of Colorado, Boulder, CO 80302, USA; Fluid-Structure Interactions Laboratory, Department of Mechanical and Industrial Engineering, University of Massachusetts, Amherst, MA 01003, USA; Fluid-Structure Interactions Laboratory, Department of Mechanical and Industrial Engineering, University of Massachusetts, Amherst, MA 01003, USA; Department of Biology, Amherst College, Amherst MA 01002, USA

## Abstract

The caridoid or “tail flip” escape behavior of decapod crustaceans is a model system in neurobiology, but many aspects of its biomechanics are not well understood. To understand how the freshwater virile crayfish *Faxonius virilis* interacts with the substrate during the tail flip, we studied tail-flip hydrodynamics and force generation for free-moving animals standing on substrate, as well as tethered animals held at different distances from the substrate. We found no significant differences in force generation when distance from substrate was varied. Particle image velocimetry revealed that vortex formation was similar at all distances, but there were notable differences in interactions between shed vortices and substrate at different distances. Negative vorticity (clockwise flow of water) was observed in tethered animals interacting with the substrate but was largely absent in free-swimming animals. We found no evidence of ground effects enhancing tail flip performance in either tethered or free-swimming individuals, as peak force generation occurred before vortex shedding. This study contributes to our understanding of the crayfish escape response and highlights the need for more work that incorporates free-swimming animals and complex environments in the study of crustacean biomechanics.

## Resumen

La reacción de escape caridoide de los crustáceos decápodos, o “coletazo,” es un sistema modelo en la neurobiología. Múltiples aspectos de su biomecánica siguen sin explicación. Para entender como el cangrejo de río de agua dulce Faxonius virilis interacciona con el substrato durante el coletazo, hemos estudiado la hidrodinámica y la generación de fuerza del coletazo en animales de movimiento libre parados sobre el substrato, al igual que en animales restringidos a varias distancias por encima del substrato. No hemos detectado ninguna diferencia significativa en la generación de fuerza cuando la distancia del substrato es variada. La velocimetría por imagen de partículas (PIV) ha revelado que la formación de vórtices es similar a todas las distancias, pero existen diferencias notables en las interacciones entre vórtices desprendidos y substrato a diferentes distancias. Hemos observado una vorticidad negativa en animales restringidos interactuando con el substrato, pero no en animales con libre movimiento. No hemos detectado que efectos relacionados al suelo aumenten el rendimiento del coletazo en individuos libres o restringidos, ya que la generación de fuerza máxima ocurrió antes del desprendimiento de los vórtices. Este estudio contribuye al entendimiento de la reacción de escape de los cangrejos de río, y destaca también la necesidad de estudios adicionales que incorporen animales con capacidad de movimiento libre y en ambientes complejos para el estudio de la biomecánica de los crustáceos.

## Introduction

Aquatic animals employ a variety of escape behaviors that are essential to their survival. Escape responses are typically rapid sequences of movements that enable animals to flee from a perceived threat. Due to their evolutionary importance and rapid speed, escape responses tend to be highly stereotyped, making them useful model systems (Feder 1972). Considerable attention has been paid to understanding the neurobiology and biomechanics underlying animal escape responses, a notable example being the C-start response characteristic of many fish (Webb 1977, 1978; Domenici and Blake 1997; Witt et al. 2015). A key assumption underlying studies of escape responses is that their performance under controlled laboratory conditions is predictive of their performance in complex natural environments (Irschick 2003). However, results from laboratory experiments often do not match those from field studies (Calisi and Bentley 2009). Irschick et al. (2005), for example, found that maximum escape speeds of lizards were not significantly correlated between field and laboratory studies. This highlights the need for laboratory approaches that more closely mimic field conditions, particularly the physical environment in which animals move. In many laboratory studies of locomotion, however, animals must be tethered or anchored in place to isolate movements of interest, or to keep animals within the view and focal range of cameras (Nauen and Shadwick 2001; van Duren and Videler 2003; Yen et al. 2003).

The caridoid escape reaction (also known as the tail flip) is an escape response exhibited by crustaceans in the order Decapoda, including lobsters, shrimp, and crayfish (Wine and Krasne 1972; Webb 1979; Heitler et al. 2000). The animal uses rapid abdominal flexion against the ventral surface of the thorax to quickly jet away, allowing it to avoid potential predators and aggressive conspecifics (Newland et al. 1988; Herberholz et al. 2004). The full caridoid response, including simultaneous limb protraction and abdominal flexion, is thought to be derived within the Malacostraca, the class containing Decapoda (Heitler et al. 2000). The tail flip is a powerful model system for understanding the evolution of neural circuitry (Edwards et al. 1999). Many of the neurons involved have no known function outside of tail flipping, and the neural circuitry involved in mediating tail flips is remarkably well-conserved across Decapoda with identical general circuitry found in roughly 50% of the 14,000 species of decapods (Faulkes 2008). Wine and Krasne (1972) characterized the two primary types of crayfish tail flips: medial giant-mediated, which are triggered by rostral stimuli, and lateral giant-mediated, triggered by caudal stimuli. Medial and lateral giant tail flips have notable differences in force generation, takeoff angle, and response latency.

Despite its long history as a model system in neurobiology, the kinematics and hydrodynamics of the tail flip have not received as much attention. In some of the first work to address this, Daniel and Meyhofer (1989) proposed squeeze forces, in which a jet of fluid is forced through the gap between the abdomen and the tail, similar to the jet-based propulsion of jellyfish, as a primary mechanism of propulsion in the shrimp *Pandalus danae*. Later, Nauen and Shadwick (2001) described the kinematics of the California spiny lobster (*Panulirus interruptus*) tail flip, finding that lobsters achieve peak tail-flip force before the tail contacts the abdomen, indicating that squeeze forces are not responsible for the majority of tail flip force generation. Rather, they found that the tail acts like a paddle to generate force. More recently, Hunyadi et al. (2020a) assessed the kinematics of crayfish tail flips, proposing a mechanism similar to that of the California spiny lobster. Using planar and volumetric particle image velocimetry (PIV), Hunyadi et al. (2020a) observed two counter-rotating vortices shed off the tips of the uropods, which lead to the formation of a horseshoe-shaped vortex that provides the propulsion for the tail flip. Prior to a tail flip, crayfish tend to raise their tail and paddle with their pleopods (Wine and Krasne 1972; Hunyadi et al. 2020a). Hunyadi et al. proposed that this pleopod motion creates a stream of fluid directed toward the tip of the tail, which is redirected by the cupped uropods to form the two vortices in the animal's wake.

Previous studies of the hydrodynamics of the tail flip were limited to animals anchored to a fixed position and elevated in the water column (Wine et al. 1975; Nauen and Shadwick 2001; Hunyadi et al. 2020a). This experimental setup makes it possible to study the free motion of the jets that are produced during the tail flip, but it fails to capture many aspects of how animals move in nature, including the interactions of vortices with the substrate and the role of ground effects in swimming performance. The ground effect is the reduction in drag and increase in lift experienced by an animal as it swims close to a flat surface (Webb 2002; Nowroozi et al. 2009). In this paper, we study the hydrodynamics of the escape response in the virile crayfish *Faxonius virilis* Hagen 1870. *Faxonius virilis* prefers rocky streams with moderate water flow, but they are also found in wetlands and lakes with sand or silt substrates and lower flow rates. *Faxonius virilis* is native to the Central United States and is invasive across much of North America and Europe. Our objectives are to quantify force generation and vortex formation in both tethered and free-swimming crayfish to better understand the role of ground effects in the caridoid escape response.

## Methods

### Study animals

Sexually mature *F. virilis* crayfish were obtained from commercial suppliers and housed communally in plastic tubs filled with 4–5 cm of aged tap water and covered with a mesh screen. The tubs were furnished with refugia (PVC pipes, terracotta flower pots, and rocks) to prevent aggressive interactions, and an air pump with an airstone to provide oxygenation. The tubs were maintained at 23–24 °C and a 14:10 light: dark cycle. Crayfish were fed algae tablets daily.

### Substrate interaction experiment

**Fig. 1:**
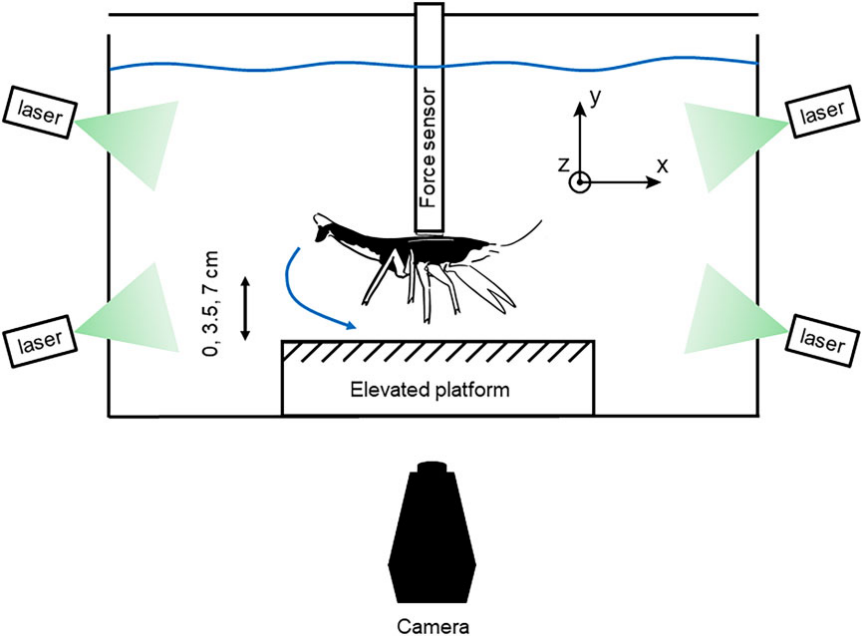
Schematic diagram of testing apparatus used in substrate interaction experiment. Within a 15 L acrylic tank, crayfish were mounted to a force sensor and suspended over a platform, which could be raised so that the platform was 0, 3.5, or 7 cm below the walking legs of the crayfish. Inset diagram shows the force axes. Crayfish were illuminated by four sheet lasers and recorded with a high-speed camera placed perpendicular to the tank.

We built a 15 L acrylic tank for substrate interaction experiments with crayfish mounted in fixed positions above a substrate (Fig. 1). The water in the tank was seeded with 10 m diameter silver coated glass spheres (Dantec Dynamics, Skovlunde, Denmark), which were illuminated by four green plane lasers (520 nm; Laserland), positioned at each corner of the front pane of the tank so that they created an illuminated plane parallel to the front of the tank. A 6-axis force sensor (Nano-17 SI-25–0.25, ATI, Apex, NJ; resolution 1/160 N and 1/32 Nmm) was positioned above the tank and anchored in place via aluminum extrusion. An adjustable Plexiglas platform, which allows for flow visualization with the lasers placed beneath the animal, was placed in the bottom of the tank and could be raised or lowered to adjust the height of the substrate. A Phantom Miro M110 high speed camera (Vision Research, Wayne, NJ, USA), set to record at 300 frames per second, was positioned in front of the tank.

Fourteen crayfish were recorded at three distances from the substrate, with three trials at each distance for each animal. Prior to experimentation, crayfish were anesthetized in ice water, and a 3D-printed plastic mount was affixed to their carapace using a combination of epoxy and cyanoacrylate. All animals were allowed at least 24 h to recover prior to testing. In each treatment, the animal was fixed in position as shown in Fig. 1, and the platform was raised to bring the substrate to the desired distance from the walking legs based on the size of the largest crayfish. In the first treatment, the adjustable platform was raised such that crayfish were 0 cm from the substrate. In the two other treatments, the platform was lowered so crayfish walking legs were suspended 3.5 cm and 7 cm above the substrate, respectively (Fig. 1). Platform heights were selected such that in the 0 cm treatment, the largest crayfish were as close as possible to the substrate without being able to touch it with their walking legs, tail, or chelae. Hereafter, these groups are referred to as 0, 3.5, and 7 cm. As shown below in our Results, these heights correspond well to the escape trajectories of free-swimming crayfish. Prior to each trial, one end of an aluminum rod was inserted into the crayfish's 3D-printed mount, and the other end was inserted into the Nano17 force sensor and secured with a set screw. The high-speed camera and force sensor were started manually, then a 7V electric shock was delivered to the antennae to induce a medial giant (MG)-mediated tail flip (Hunyadi et al. 2020a). Anomalous tail-flips, such as those where crayfish struck the substrate during their tail flip, were eliminated, leaving a total of 127 individual tail-flip events trials. Due to mortality over the course of the experiment, not all animals were able to be tested at all three distances.

The data recorded on the force sensor were loaded into MATLAB version 2021a for analysis (Mathworks Inc., Natick, MA, USA). The magnitude and direction of the force vector generated by each animal was calculated from the measured force components in the *x*- and *y*-directions. For each tail-flip event, the peak force and the corresponding angle at which peak force occurred were either measured directly from the time series (for single tail flips) or taken as an average across the peaks (for tail flips occurring in rapid succession). Variance between tail-flip treatments was compared using Bartlett's test, and normality was assessed visually using histograms and the Shapiro–Wilk test. Differences in peak tail-flip force generation and peak force angle were then assessed using linear mixed effects models with force or angle as fixed effects and identity of the crayfish as a random effect. Mixed effects models were generated using the lme4 package for R (Bates et al. 2015). Coefficients of variation were calculated as the ratio of the standard deviation (SD) to the mean and provide a normalized representation of the extent of variation around the mean.

Flow visualization for the tail-flip videos were generated using PIVLab, an open-source MATLAB plugin for PIV analysis (Thielicke and Stamhuis 2014, Thielicke and Sonntag 2021). Analysis on this set of experiments was performed using the default PIVLab parameters, and an integration window size of 70 × 70 pixels and 50 × 50 pixels for the first and second passes, respectively. PIV requires a mask to be generated to cover foreground objects in each frame that is analyzed. All masks for the visualizations presented in the results were manually drawn, while masks for the PIV videos included in the supplementary materials were generated automatically using a custom-built program.

### Free-swimming crayfish experiment

**Fig. 2:**
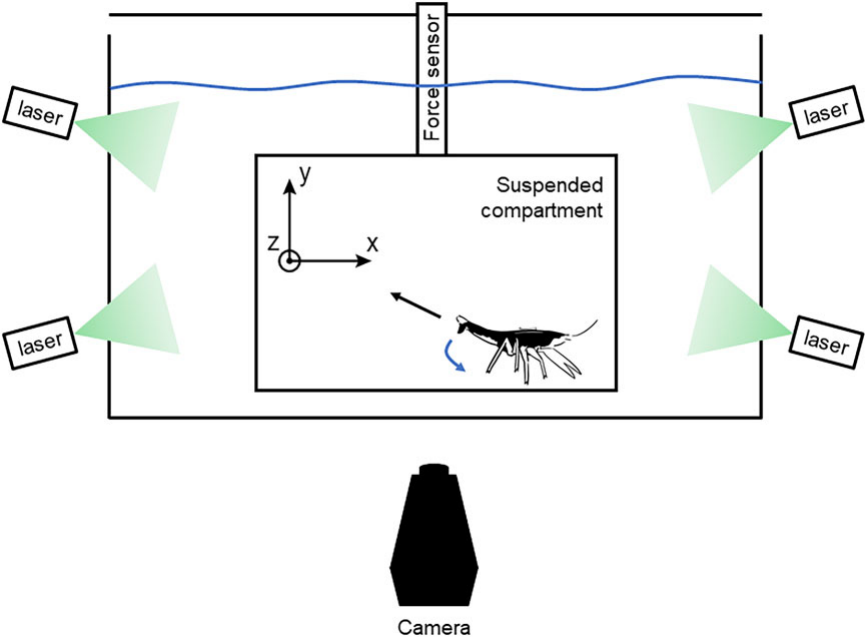
Schematic diagram of testing apparatus used in the free-swimming crayfish experiment. Within a 37.5 L glass tank, a transparent compartment 22 cm wide, 14 cm high, and 10 cm deep was attached to a force sensor and suspended in the water. Within this compartment, the initial trajectory of the tail flip could proceed unimpeded. Inset diagram shows the force axes. Crayfish were illuminated by four sheet lasers and recorded with a high-speed camera placed perpendicular to the tank.

A second, larger apparatus was assembled to record tail flips in free-moving animals. The apparatus consisted of a 25 × 30 × 50 cm, 37.5 L glass aquarium, which contained a suspended compartment affixed to a Nano-17 force sensor (Fig. 2). The top and bottom of the compartment were Plexiglas panels, rigidly attached to one another with metal screws, and the sides were covered with transparent plastic film to prevent animals from moving off the sides of the lower platform. The front and back of the compartment were left open.

Five crayfish were recorded in 10 total trials in the free-swimming experiment, with each individual being tested 1–3 times. None of these crayfish were previously used in the substrate interaction experiment. Crayfish were held above the chamber floor by hand until the force recording was started. They were then dropped onto the substrate and allowed to walk forward until their antennae were positioned between the stimulator wires. A 7V electric shock was delivered, resulting in a tail flip, and the camera was triggered manually. Videos were aligned to the force sensor data by tapping the force sensor in the frame of the video.

Flow visualizations were once again generated using PIVLab, this time with three integration passes using windows of 50, 35, and 20 pixels square. Crayfish positions were tracked over the course of the tail flip using Tracker, a program for object tracking and video modeling (Brown 2012). The approximate center of mass (COM) of crayfish was manually identified in each frame of the tail-flip videos. The position data were then loaded into R version 4.3.0 to calculate tail-flip trajectories and takeoff angles (R Core Team 2023). The *x*- and *y*-coordinates of the animals were standardized relative to each animal's starting position. Takeoff angles (${\theta}$) were calculated as


\begin{eqnarray*}
\theta = \textit{arctan}\!\left({\frac{{\Delta y}}{{\Delta x}}} \right).
\end{eqnarray*}

Means are reported ± SD, and differences are considered statistically significant if *P* < 0.05. All statistical analyses were performed in R Version 4.3.0 using Tidyverse packages (Wickham 2016, 2019, 2023; R Core Team 2023).

## Results

### Substrate interaction experiment

**Fig. 3:**
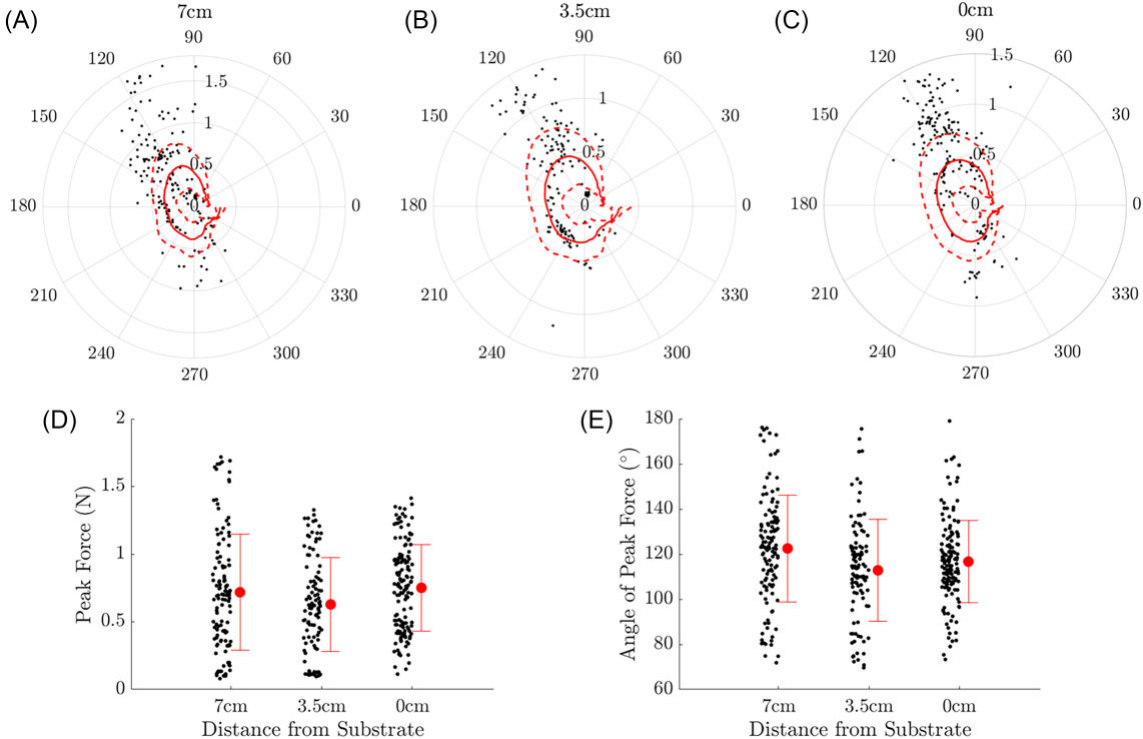
Force sensor data for all trials. Panel A shows a scatter plot (black dots) of the local maxima of force generated and the corresponding angle, as well as the mean force at each angle (solid line) and ±1 SD (dotted lines) for the 7 cm group. Panels B and C show the same for the 3.5 cm and 0 cm groups, respectively. Panels D and E show jitter plots of the force maxima and corresponding angle respectively from the thrust generating stage of the tail flips, along with the mean value and standard deviation.

Crayfish used in this experiment had a mean length of 8.66 ± 0.75 cm. Peak force produced by the tail flip in single tail flip events did not differ significantly based on the height of the crayfish over the substrate (linear mixed effects model: *r*^2^ = 0.84, *P*_7-3.5_ = 0.28, *P*_7-0_ = 0.80, *P*_3.5–0_ = 0.36; Fig. 3D). Likewise, the angle of the force vector at the moment of peak force generation (peak force angle) did not differ significantly among treatments (linear mixed effects model: *r*^2^ = 0.24, *P*_7-3.5_ = 0.76, *P*_7-0_ = 0.72, *P*_3.5–0_ = 0.53; Fig. 3E). The coefficients of variation for force and angle within each treatment group were as follows: CV_F7_ = 0.392, CV_A7_ = 0.232, CV_F3.5_ = 0.532, CV_A3.5_ = 0.206, CV_F0_ = 0.355, and CV_A0_ = 0.462. Overall the coefficients of variation for force and angle were CV_F_ = 0.404 and CV_A_ = 0.329, respectively, indicating similar variation between individuals within treatment groups as across treatment groups. Forces and peak force angles for single tail flip events, as well as means and medians, are shown in Supplementary Table S1A–C. In some cases, crayfish performed double tail flips or a series of tail flips in rapid succession; polar plots of these are shown in Fig. S1–S3. The peak force generated in a series of tail flips is relatively constant from flip to flip, and comparable to the peak force and corresponding angle recorded for single flip events. There is a significant difference in the time series of force data at the end of a tail flip in a series of flips, however, where the re-extension of the tail in anticipation of the successive tail flip generates a downward force on the animal. These appear as the local maximum of force in the lower quadrants of the polar force plots in Fig. 3A–C, while the local maximum of force shown in the upper two quadrants corresponds to the thrust generating portion of the tail flip. By averaging the force generated at each angle in the tail flip cycles for all trials as shown in red in Fig. 3A–C, we can see that there is a strong similarity in the patterns of force generated at all three substrate heights. Note that the average forces shown in Fig. 3A–C are less than the mean peak force generated as shown in Fig. 3D, which shows the mean of peak force independent of the specific angle at which it occurs.

**Fig. 4:**
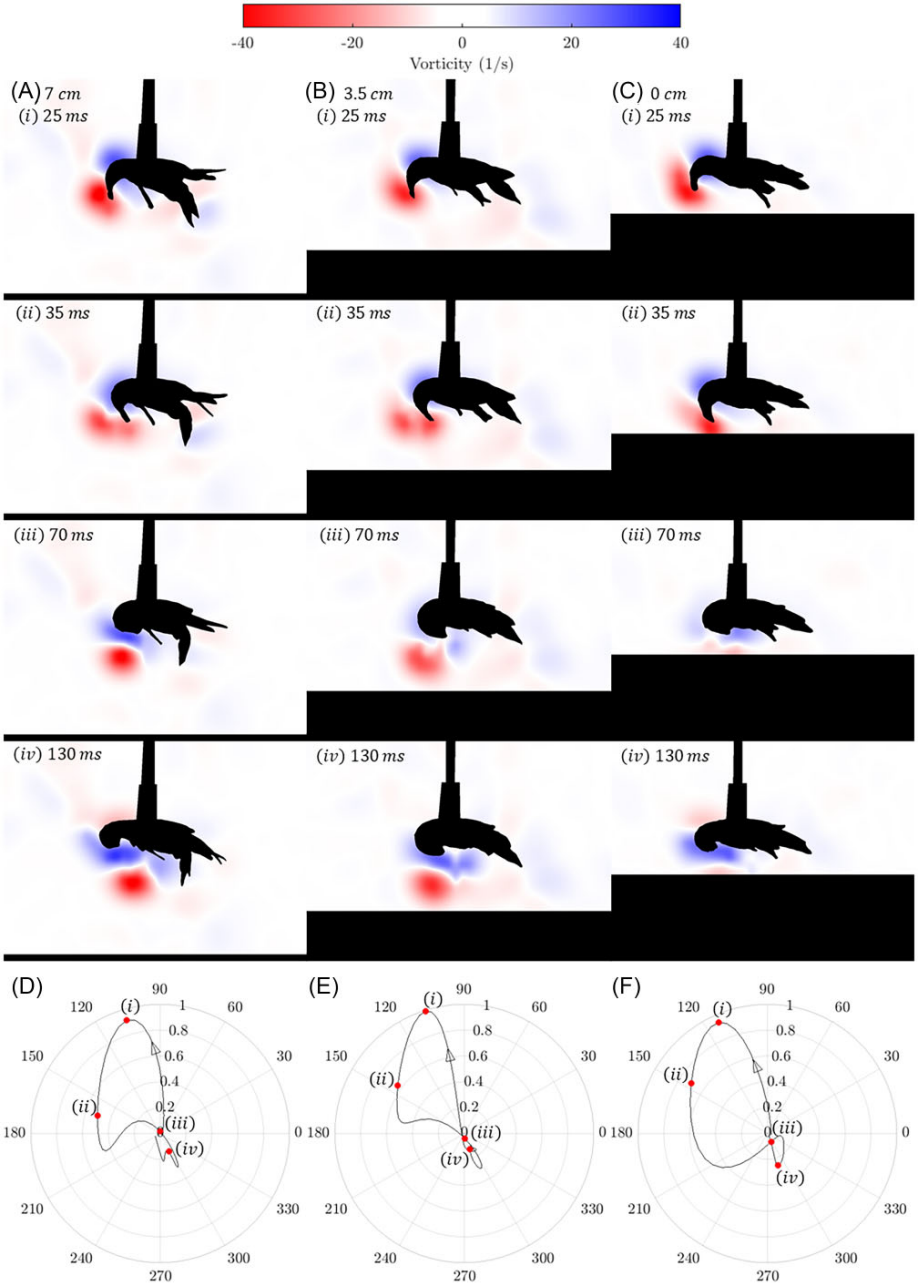
Flow visualizations of a tail flip by a mounted crayfish. The cases shown here are all from one animal but are representative of all tail flips observed. Columns are for (A) 7 cm, (B) 3.5 cm, and (C) 0 cm above the substrate. Row (i) corresponds to the moment of maximum force generated (about 25 ms after start of tail flip for all cases), and rows (ii), (iii), and (iv) show 35 ms, 70 ms, and 130 ms after the start of the tail flip respectively. Polar plots (D-F) at the bottom show magnitude and orientation of force generated in Newtons and degrees for the single tail flip event shown in the flow visualization. Note that the difference in force generated at the three heights for the individual shown here is less than the mean difference in forces for all individuals shown in Fig. 3A–C. Arrowhead indicates direction of increasing time and dots show positions of corresponding frames along the force trajectory.

In the flow visualization images of Fig. 4A–C rows (i) and (ii) we see that the development of the vortex structure, a three-dimensional ring that appears in the 2D PIV images as a pair of counter rotating vortices, remains effectively the same at all heights above the substrate. As discussed in Hunyadi et al. (2020a), the vortex ring is formed on the dorsal side of the tail, with positive and negative regions of vorticity forming at the tail-body connection and the posterior tip of the tail, respectively. he moving fluid pushed by the tail interacts with the surrounding still fluid and is forced out radially away from the direction of motion of the jet, creating the rotating region of flow that is shown in the PIV images. In Fig. 4A–C row (iii) the tail has contacted the abdomen and the vortex ring and accompanying jet have been shed from the outside of the tail down toward the substrate and anteriorly toward the crayfish's head. At this point, the influence of the proximity of the substrate on the structure of the shed vortices becomes apparent.Figure 4C(iii) shows that at the nearest distance (0 cm) the vortex ring is immediately disrupted through contact with the substrate, and while there is still some cohesive flow moving anteriorly toward the head of the crayfish, the negative vortex (negative indicating that the region of fluid rotates in a clockwise direction from the observer's perspective) seen in Fig. 4A(iii) and 4B(iii) is not present. There is still some evidence of a positive vortex below the crayfish, corresponding to the upper part of the vortex ring, indicating that the ring breaks at the bottom first. It should be noted that the positive vortex, visible in Fig. 4C(iv), is associated with the re-extension of the tail and is not a remnant from the jet produced during the tail flip. Vortex patterns were similar across all animals.

When examining the force sensor data shown in Fig. 4D–F, it is apparent that the peak force in the tail flip is generated, while the vortex is attached to the tail and occurs at frame (i) of the visualizations. The substrate, however, does have a substantial influence on the structure of the shed vortex ring between frames (iii) and (iv), where the force plots show minimal forces generated, indicating that the changes in the vortex rings due to their interactions with the substrate do not contribute to variance in force generation at different substrate heights. Videos corresponding to Fig. 4 can be found in the Supplementary videos S1A–C.

### Free-swimming crayfish experiment

**Fig. 5:**
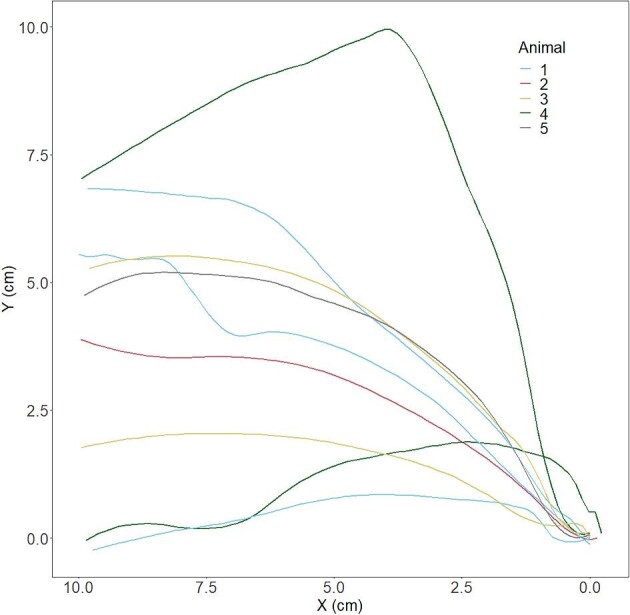
Trajectory plot of takeoff and swimming trajectories after a tail flip. Lines represent nine tail flips performed by five crayfish; each crayfish is represented 1–3 times. The tenth trial was excluded since the crayfish collided with the force sensor. Axes show the *x*- and *y*-position (in cm) of crayfish relative to their starting position (bottom right corner).

**Fig. 6:**
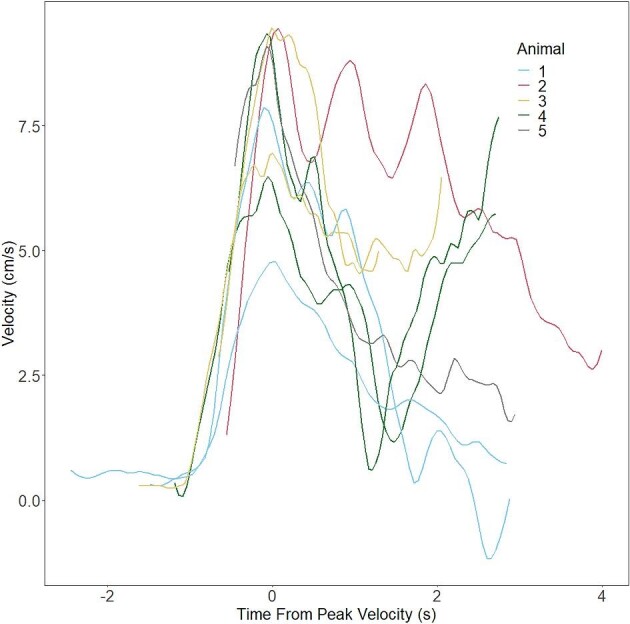
Velocities of free-swimming crayfish after takeoff from substrate. Lines represent nine flips performed by five crayfish; each crayfish is represented 1–3 times. Axes show the time relative to the moment of peak velocity, and the velocity in cm/s.

The crayfish used in this experiment had a mean body length of 8.62 ± 0.35 cm. When induced to tail flip directly from the substrate in the tank (Fig. 2), crayfish produced a mean magnitude of forces of 8.99 ± 3.10 N (median = 1.63 N). The mean takeoff angle was 140.29 ± 4.80° (*n* = 5 crayfish). Figure 5 shows the trajectories from free-swimming crayfish following takeoff, smoothed using a LOESS function; an additional plot of displacement over time can be found in Fig. S4. The initial propulsion sends the animal on an upward arc, the specific path of which varies not only from crayfish to crayfish, but also among tail flips by the same animal. Crayfish achieved a mean peak velocity of 9.15 ± 0.29 cm/s (median = 9.43 cm/s). Figure 6 shows the velocities of crayfish over time in this experiment. Data are aligned such that the peak velocity is achieved at time *t* = 0.

**Fig. 7:**
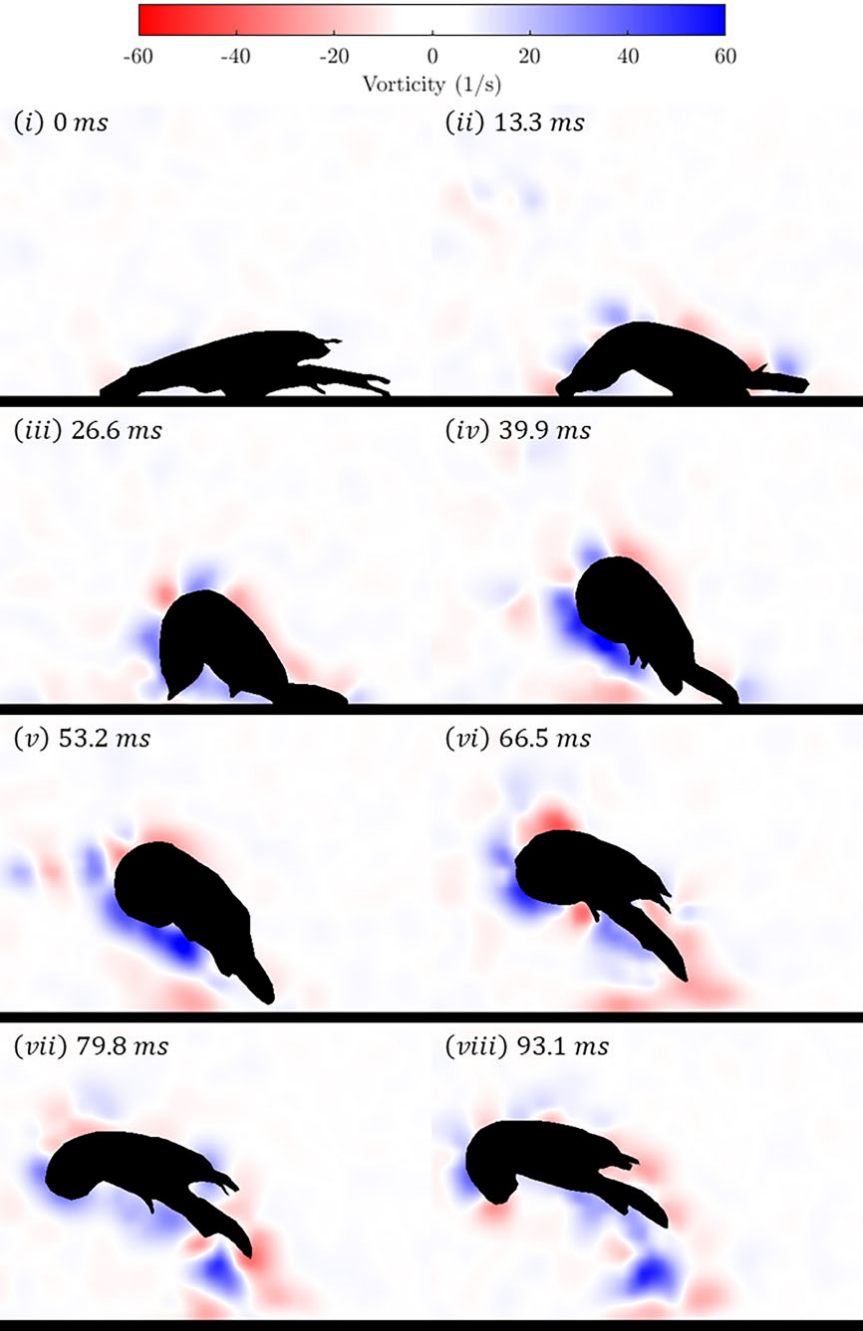
Flow visualization of a free-swimming crayfish performing a tail flip from the substrate. Panels are 13.3 ms apart. Unlike the substrate interaction results shown in Fig. 4, there is no significant development of negative vorticity during the tail flip. Instead, the only coherent, persisting vortex structure is the positive vortex forming at the tail-body connection.

We conducted PIV flow visualization for a sample case of these tail flips. Figure 7(i) shows the moment immediately before the tail flip begins, and by Fig. 7(iv) the tail flip is complete. The entire tail flip occurred in approximately 40 ms, and for the next 40 ms or so the crayfish maintained contact between its tail and abdomen, while coasting on the momentum provided by the initial tail flip (v–vi). At this point the crayfish began to extend its tail (vii–viii), and, after straightening out its tail parallel to the substrate, the crayfish tail flipped 1–5 more times while suspended in the water column, continuing to propel itself away from the source of the stimulus. These subsequent tail flips were not within the plane of the lasers, so fluid flow could not be visualized. Video corresponding to Fig. 7 can be found in Supplementary video S2. PIV videos for two additional free-swimming individuals are also included in the Supplementary videos S3–4.

## Discussion

In our analysis of tail flips performed by the virile crayfish (*F. virilis)*, we found no significant relationship between force generation and takeoff angle of the tail flip, and no differences in force generated depending on the distance of the animal from the substrate. However, flow visualizations revealed major differences in hydrodynamic interactions between free-swimming crayfish and crayfish mounted in a fixed position, and between crayfish mounted at varying distances from the substrate.

Tail flips at near, intermediate, and far distances produced similar forces, likely due to the mechanism of force generation. Crayfish achieved their peak force before the uropods—which along with the telson form the tail fan (Hunyadi et al. 2020a)—were fully closed against the abdomen, and before the vortices were shed. The vortices did not interact with the substrate until after this point, and at this point had a minimal effect on force generation. This behavior is similar to that documented in California spiny lobsters by Nauen and Shadwick (2001), which also achieve peak force generation prior to closing the tail fully against the abdomen. Our study provides strong evidence that this mechanism drives crayfish swimming in the water column and suggests that it may be applicable to other decapod crustaceans across a range of body sizes.

The formation of a vortex ring anteriorly to crayfish during the tail flip was previously observed in PIV results from Hunyadi et al. (2020a), and the vortex ring breakup mechanism observed in Fig. 4C has been documented in previous studies examining vortex rings colliding with inclined surfaces (Lim 1989; New et al. 2016). For a wide variety of contact angles, these studies show that when the vortex ring breaks, there is a formation of bi-helical vortex lines. As the ring breaks, the disruption propagates around the vortex ring creating ripples and secondary vortices that coalesce at the leading edge of the vortex ring. While not directly observable from the 2D flow visualizations shown here, these secondary vortices have dissipative effects that cause the positive vortex to weaken as it moves anteriorly.

Unlike the cases shown in Fig. 4C, the positive vortex observed in the free-swimming animal in Fig. 7 persists in time, implying that there is minimal, if any, formation of secondary vortices. In Fig. 7(ii) there is a small negative vortex formed at the tip of the tail, but this does not persist throughout the tail flip. Instead, a small negative vortex forms along the substrate. In their study of vortex rings interacting with inclined surfaces, New et al. (2016) observed that at steeper contact angles, the part of the vortex ring that first contacts the surface mostly dissipates, although a small portion is entrained between the remainder of the vortex ring and the surface. In these instances, the bi-helical vortex lines do not form and the portion of the vortex ring further from the surface persists. It would seem therefore that a tail-flip event beginning on a surface creates a flow field that is similar to a vortex ring contacting a surface at a steep angle, in spite of the fact that no vortex ring is fully formed, instead it appears that the only significant vortex present is that formed at the tail-body connection. This similarity between the flow generated by tail-flips of free-swimming crayfish and that of a vortex ring contacting a surface at a steep angle could inform future modeling of the tail-flip.

Our PIV analysis showed no evidence of ground effects enhancing tail flip performance in either tethered or free-swimming individuals, as peak force generation occurred before vortex shedding. Ground effects, the reduction in hydrodynamic drag on propulsors operating near a solid boundary, are well characterized in animals moving parallel to the surface, but “unsteady” effects of oscillating or undulating propulsors are less understood (Quinn et al. 2014). The magnitude of the ground effect is negative related to the gap/span ratio (*z*/*B*), where the gap is the distance between the thrust-producing structure and the substrate, and the span is the linear measurement of the thrust-producing structure (e.g., body depth in Pleuronectiformes fish, wingspan in Rajiformes fish, uropod width in some Decapoda crustaceans). Previous work has shown that the ground effect is minimal for axial undulatory swimming at *z*/*B* ≅ 2, and for rigid bodies, similar to those of crayfish, at *z*/*B* ≅ 3 (Webb 2002). Similarly, Su et al. (2022) argue that ground effects become negligible at *z*/*B* ≅ 3. We did not collect detailed morphological data as part of the current study, but a previous study using similar *Faxonius* crayfish found that the total combined width of the uropods was 35.40 ± 2.51 mm (Hunyadi et al. 2020b). Thus, if our crayfish performed tail flips with the uropods fully extended, the maximum *z*/*B* in the current study (*h* = 3.5 or 7 cm) would be ≅1–2. If the uropods were retracted (the mean width of the central telson is only 9.45 ± 0.99 mm, Hunyadi et al. 2020b) these values would more than double. The lateral positioning of our camera did not allow us to determine whether the uropods were extended or retracted during tail flips, but these estimates based on morphology (i.e., gap/span ratios) suggest that ground effects were minimal, especially at the greatest height we used (*h* = 7 cm) and are thus consistent with our PIV results.

Studies of ground effects in crustaceans are rare, but there is a rich literature on escape behavior in benthic fish. Flatfishes (Pleuronectiformes) swim on their sides and at rest lie in full contact with the substrate (Webb 1981, 2002). In their fast-start take-offs, the flatfish body forms a U-shape, pushing off from the substrate with the medial section of their bodies, which contrasts with the inverted U-shape of the crayfish body as it pushes off with its tail fan (see Fig. 7[iii]). Webb (1981) studied the kinematics of the fast start in speckled sanddab (*Citharichthys stigmaeus*) resting on mesh screens 0, 1, 3, and 6 cm from the true bottom, and found that distance to the true bottom had no impact on the velocity of the fast start (*x*- and *y*-displacement of the COM), which suggested that distance to the substrate provided no hydrodynamic benefit. In a subsequent study, Webb (2002) investigated the hydrodynamic benefit of the ground effect for another flatfish (plaice *Pleuronectes platessa*) swimming at heights (*h*) of 0, 10, 50, or 100 mm above the true bottom of the tank, which resulted in gap/span ratios (*z*/*B*) ranging from 0.23 (*h* = 0) to 2.16 (*h* = 100 mm). Tailbeat frequencies increased with swimming speed in plaice swimming near the bottom, but showed no relationship at *h* ≥ 10 mm. Perhaps most importantly, the power contribution of the tail increased monotonically with *z*/*B* until a value of ≅1.1 (Webb 2002). Ground effects are likely important in other groups of benthic fishes (e.g., Webb 1981; Nowroozi et al. 2009; Ogunka et al. 2002, 2020), though their modes of swimming may have less in common with the crustacean caridoid response.

Much of our previous understanding of the caridoid escape response in crustaceans comes from laboratory conditions in which animals are partially restrained (Newland and Neil 1990; Nauen and Shadwick 2001; Hunyadi et al. 2020a). In one notable exception, Newland and Neil (1990) studied free-swimming Norway lobsters (*Nephrops norvegicus*) and described kinematically distinct tail flips depending on whether the escape response was initiated due to mechanical stimulation of the rostrum (anterior) or telson (caudal/posterior). Rostral stimuli induce tail flips via the MG axon and caudal stimuli induce tail flips via the lateral giant (LG axon) (see also Heitler et al. 2000). With the exception of one outlier (Fig. 5), our mean takeoff angle of ∼140° for rostral-MG tail flips in *Faxonius* crayfish was consistent with the rostral-MG tail flips in *N. norvegicus*, in contrast to the ∼110° they observed for telson-LG tail flips (Newland and Neil 1990).

The caridoid, or tail flip, response is crucial to crayfish survival and evolutionary fitness, as it enables them to escape predators and aggressive conspecifics (Newland et al. 1988; Herberholz et al. 2004). Many species of crayfish, including *F. virilis*, are invasive in many parts of the world (Gherardi et al. 2011). Management strategies include the enhancement of predatory fish populations via restriction of recreational fishing permits, which results in an increase in fish predation on juvenile crayfish (Bajer et al. 2019). Thus, an understanding of the crayfish escape response—particularly the swimming trajectories of crayfish in unrestrained conditions—could provide insight into the effectiveness of such biocontrol strategies. This paper sheds light on substrate effects during the tail flip and presents the first use of PIV on free-swimming crayfish, bringing to light the complex hydrodynamics associated with this movement. Crayfish do not gain a mechanical advantage by swimming at any certain distance above substrate, so any preference for where in the water column to swim must be driven by other factors, such as predation risk, currents, or avoiding benthic obstacles. This work will provide a foundation for future studies of decapod swimming, especially relating to behavior and ecology.

## Supplementary Material

obae027_Supplemental_Files

## Data Availability

Data and R code will be available in the Supplementary Materials.
