# Supplementary material for: Substrate Interactions and Free-Swimming Dynamics in the Crayfish Escape Response
Source: Integr Org Biol. 2024 Jul 12;6(1):obae027. doi: 10.1093/iob/obae027 (PMC11288282; doi:10.1093/iob/obae027)
Supplement: obae027_Supplemental_Files [file obae027_supplemental_files.zip › Supplementary Figures.pdf]

Supplementary figures

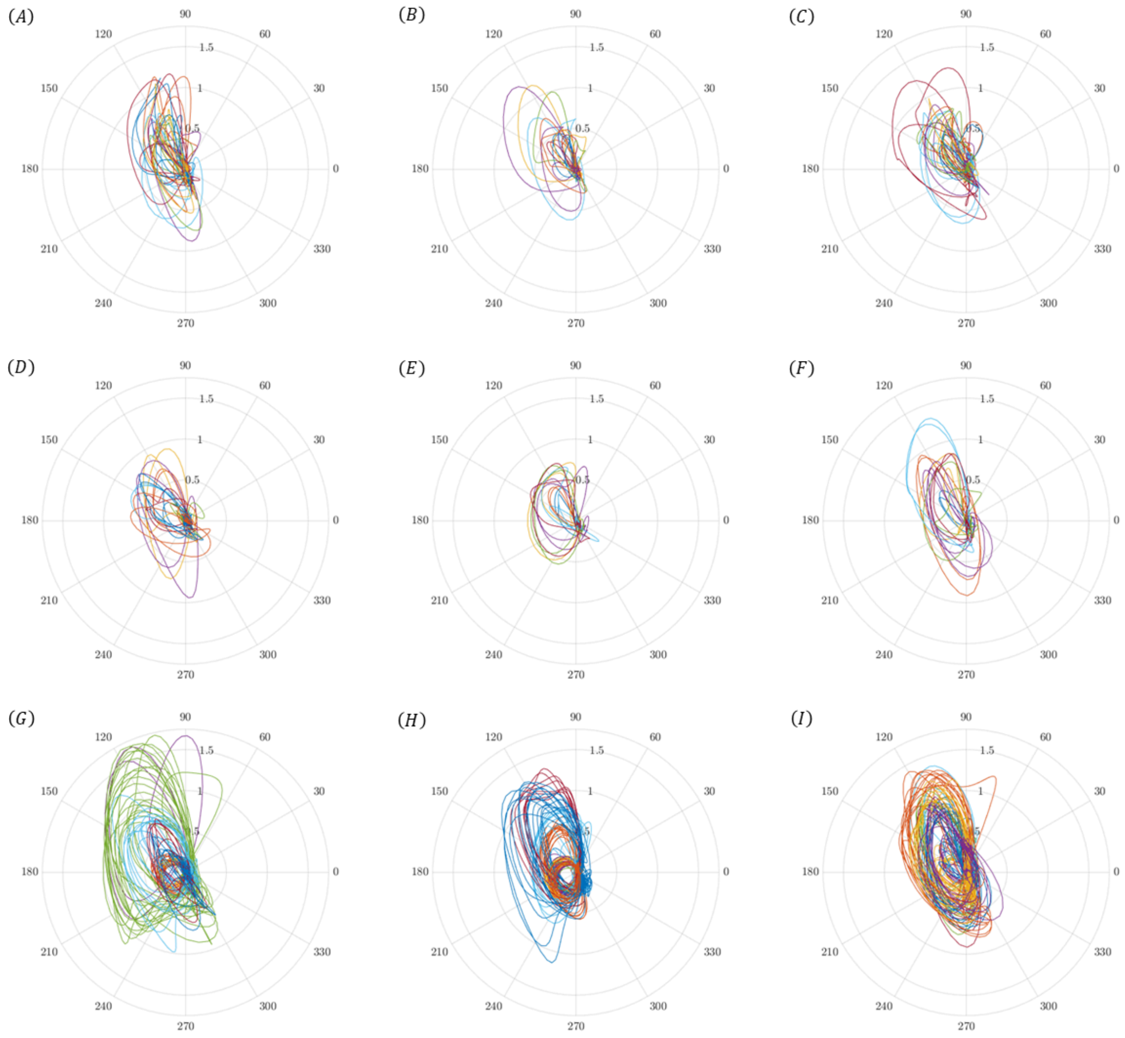

Figure S1. Polar plots showing single tail flips at (A) 7 cm, (B) 3.5 cm, and (C) 0 cm. Double flips at (D) 7 cm, (E) 3.5 cm, and (F) 0 cm. Series flips at (G) 7 cm, (H) 3.5 cm, and (I) 0 cm. All tail flip events are shown.

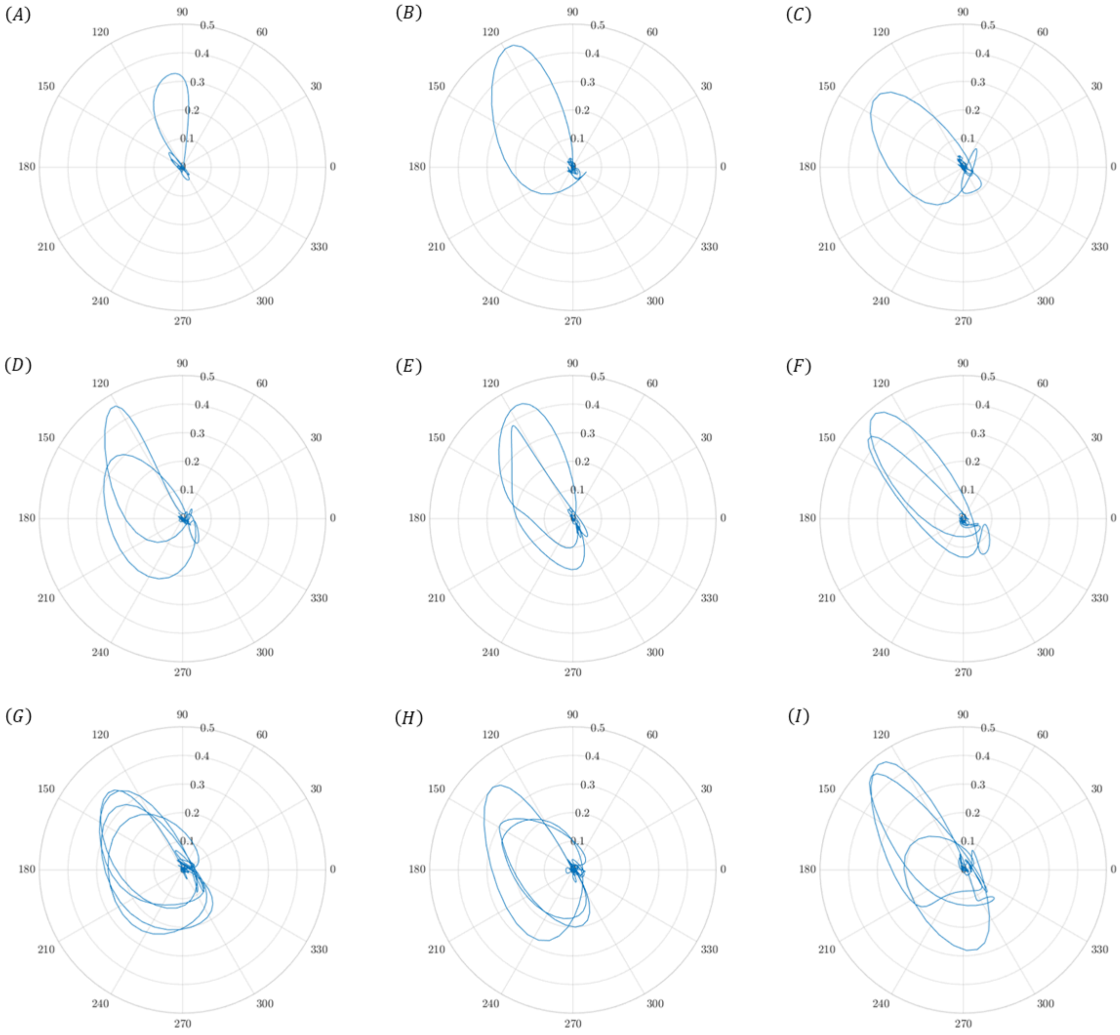

Figure S2. Polar plots showing single, double, and series tail flips for one crayfish. Single tail flips at (A) 7 cm, (B) 3.5 cm, and (C) 0 cm. Double flips at (D) 7 cm, (E) 3.5 cm, and (F) 0 cm. Series flips at (G) 7 cm, (H) 3.5 cm, and (I) 0 cm.

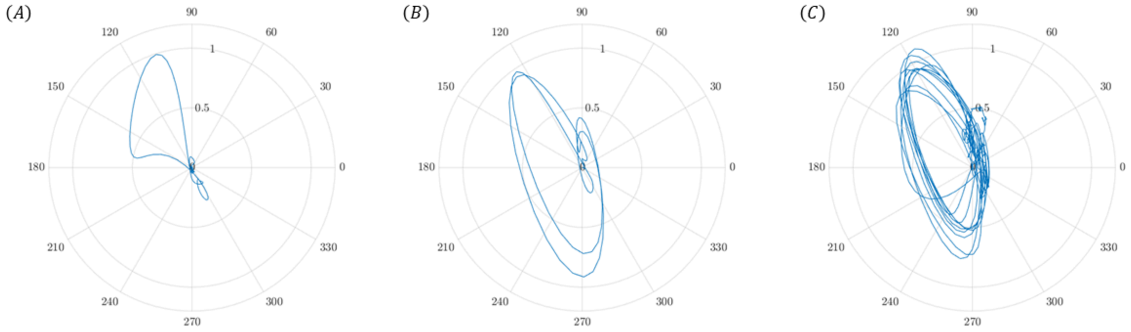

Figure S3. Polar plots showing a typical single tail flip (A), double flip (B), and series flips (C) from one crayfish (same animal as Fig. 4.). In double and series flips, re-extending the tail creates force opposite to the main thrust, ~270 degrees compared to ~120 degrees. It would seem some work is undone by flipping more than once, however this is only the case when animals are fixed in place. In the videos of the free-swimming cases, it is clear that successive flips are not as counterproductive as these plots would imply.

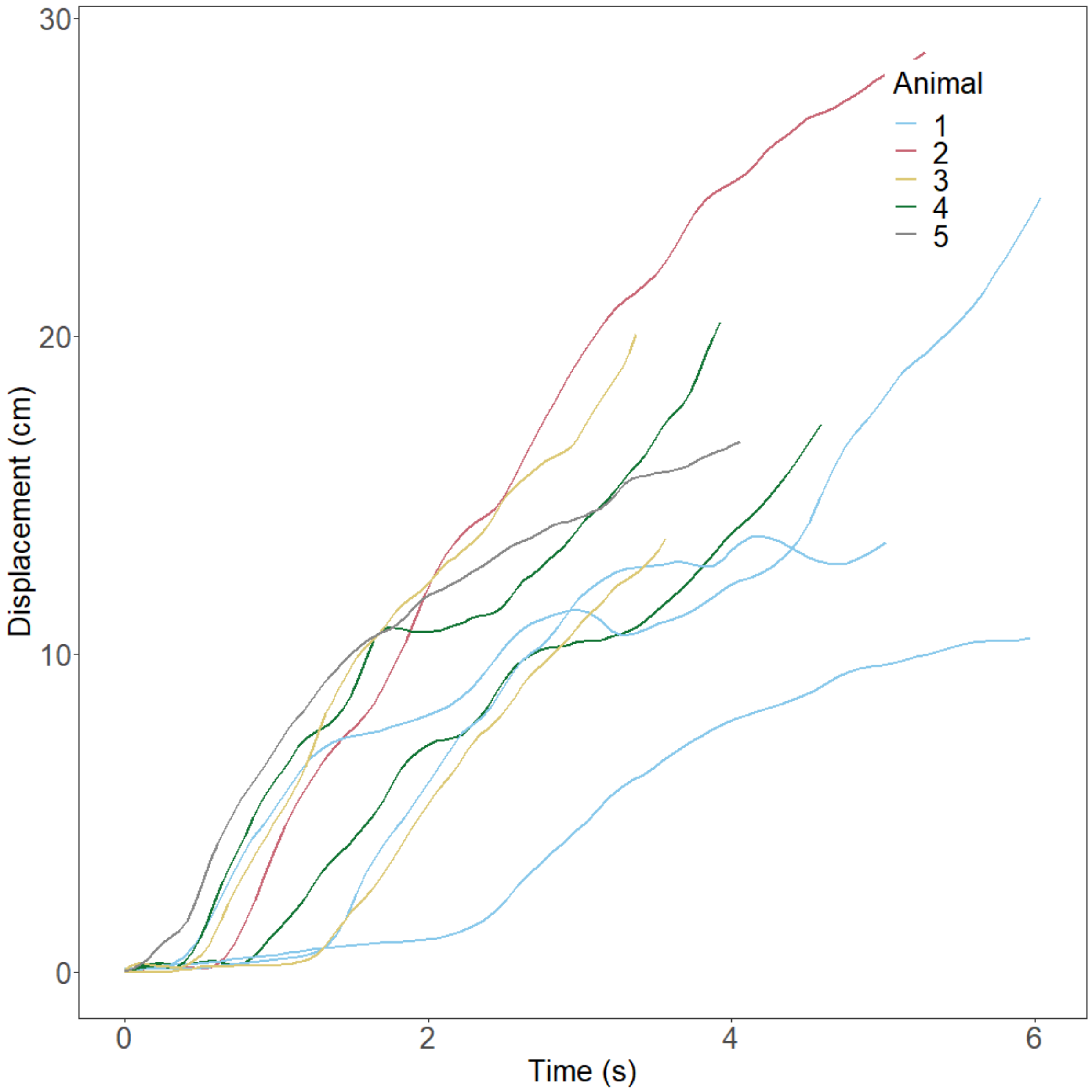

Figure S4. Euclidean distance of free-swimming crayfish from their starting location after a tail flip.
